# Essential role of GATA3 in regulation of differentiation and cell proliferation in SK-N-SH neuroblastoma cells

**DOI:** 10.3892/mmr.2014.2809

**Published:** 2014-10-29

**Authors:** HONGWEI PENG, XIAO-XUE KE, RENJIAN HU, LIQUN YANG, HONGJUAN CUI, YUQUAN WEI

**Affiliations:** 1 Laboratory of Cancer Biotherapy, State Key Laboratory of Biotherapy, West China Hospital, Sichuan University, Chengdu, Sichuan 610041, P.R. China; 2 State Key Laboratory of Silkworm Genome Biology, Southwest University, Chongqing 400716, P.R. China

**Keywords:** cell proliferation, differentiation, GATA3, neuroblastoma

## Abstract

Neuroblastoma is a common solid malignant tumor of the sympathetic nervous system, which contributes to 15% of cancer-related mortality in children. The differentiation status of neuroblastoma is correlated with clinical outcome, and the induction of differentiation thus constitutes a therapeutic approach in this disease. However, the molecular mechanisms that control the differentiation of neuroblastoma remain poorly understood. The present study aimed to define whether GATA3 is involved in the differentiation of neuroblastoma cells. The results demonstrated that GATA3 is a prognostic marker for survival in patients with neuroblastoma, and that high-level GATA3 expression is associated with increased self-renewal and proliferation of neuroblastoma cells. Retinoic acid treatment led to GATA3 downregulation together with neuronal differentiation, suppression of cell proliferation and inhibition of tumorigenecity in neuroblastoma cells. These findings suggest that GATA3 is a key regulator of neuroblastoma differentiation, and provide a novel potential therapeutic strategy for the induction of neuroblastoma differentiation.

## Introduction

Neuroblastoma is a common childhood malignant tumor of the sympathetic nervous system, accounting for up to 10% of pediatric cancers and 15% of cancer-related mortality in children (1–3). Neuroblastoma comprises a heterogeneous group of tumors, in which the level of differentiation is known to be of prognostic significance (4,5). Histologically, neuroblastomas range from tumors containing poorly-differentiated neuroblasts to those composed of fully-differentiated sympathetic neurons (6,7). Patients with poorly differentiated neuroblastomas have a significantly poorer survival than those with neuroblastomas that are shown to be well-differentiated on histological examination (4,5,8).

Retinoic acid (RA) is an effective inducer of the differentiation of neuroblastoma cells (9,10), which has been used in clinical practice as a therapeutic agent in high-risk neuroblastomas in order to improve the differentiation state of the cells (11,12). In addition, GATA transcription factors are involved in the regulation of hematopoiesis, and the development of the cardiovascular, nervous, and urogenital systems (13–17). The GATA family contains six members, which are reported to be expressed in distinct spatiotemporal patterns (18–20). GATA2 and GATA3 are the only members of this family that are present in the nervous system (21,22), and the pattern of the expression of these two proteins is known to overlap. GATA3 has been reported to be involved in the development of serotonergic neurons during formation of the ear, and in the development of the caudal raphe nuclei and the peripheral nervous system (22–27). The present study investigated the role of GATA3 in neuroblastoma proliferation and differentiation.

## Materials and methods

### Cell culture

SHEP1, SK-N-DZ, SK-N-AS, and SK-N-SH human neuroblastoma cells were grown in Dulbecco’s modified Eagle’s medium (DMEM) supplemented with 10% fetal bovine serum (FBS). SK-N-BE (2), IMR32, BE (2)-C and SY5Y human neuroblastoma cell lines were grown in a 1:1 mixture of DMEM and Ham’s nutrient mixture F12 (F12/DMEM), supplemented with 10% FBS and nonessential amino acids. LAN-6 and SMS-KCNR human neuroblastoma cell lines were grown in RPMI-1640 supplemented with 10% FBS. The growth media and FBS were obtained from Invitrogen Life Technologies (Carlsbad, CA, USA). All cells were obtained from American Type Culture Collection (Manassus, VA, USA) and cultured at 37°C in a 5% CO_2_ humidified incubator. The 293GPG retroviral packaging cell line was cultured as described previously (28).

### Retroviral production and infection

The retroviral constructs, pBabe-green fluorescent protein (GFP) and pBabe-GATA3, were used in the overexpression experiments. Retroviruses were produced using the 293GPG packaging cell line as described previously (28). At 24 h following the final round of retroviral infection, cells were cultured in the growth medium containing 1.0 μg/ml puromycin for three days, and drug-resistant cells were pooled. The percentage of retrovirus-infected cells ranged between 80 and 90%, as estimated in parallel infections using the retrovirus-expressing GFP. Over-expression of relevant proteins was verified by an immunoblotting assay.

### Immunoblot analysis

Following RA (Sigma-Aldrich, St. Louis, MO, USA) treatment or retroviral infection, cells in the exponential growth phase at 70–80% confluence were harvested at various time points and washed once with ice-cold phosphate-buffered saline. Cell pellets were suspended in SDS sample buffer and boiled for 10 min prior to centrifugation at 211 × g for 10 min. Samples were subjected to 12% SDS-polyacrylamide gel electrophoresis (SDS-PAGE) and transferred to a polyvinylidene fluoride membrane (EMD Millipore, Billerica, MA, USA). The membrane was probed with antibodies and binding was visualized using enhanced chemiluminescence (ECL; Beyotime Institute of Biotechnology, Haimen, China). The following primary antibodies were used: Rabbit polyclonal anti-GATA3 (1:100; H-48, sc-9009; Santa Cruz Biotechnology Inc., Dallas, TX, USA), mouse monoclonal anti-Mash1 (1:100; clone 24B72D11.1; BD Pharmingen, San Diego, CA, USA), rabbit polyclonal anti-peripherin (1:2,000; AB1530; Chemicon International, Inc., Billerica, MA, USA) and mouse monoclonal anti-α-tubulin (1:10,000; B-5-1-2; Sigma-Aldrich). Horseradish peroxidase-conjugated goat anti-mouse and goat anti-rabbit IgG (1:5,000, ICN, Bryan, OH, USA) were used as secondary antibodies.

### Cell growth and differentiation assays

For differentiation assays, RA was dissolved in dimethyl sulfoxide (DMSO) and 10 mM stock solutions were prepared. SK-N-SH cells were treated with 1 μM RA. DMSO (0.1%; Sigma-Aldrich) was used as negative control. Cell growth was observed under a microscope (Olympus IX71; Olympus, Tokyo, Japan) and determined by MTT analysis (Sigma-Aldrich)

### Patient data analysis

Patient data and gene expression datasets were obtained from the Oncogenomics Section Data Center (http://pob.abcc.ncifcrf.gov/cgi-bin/JK). Kaplan-Meier analysis and resulting survival curves were created using GraphPad Prism (version 6.0; GraphPad Software, Inc, La Jolla, CA, USA). All data and P-values (log-rank test) for these experiments were downloaded online (http://pob.abcc.ncifcrf.gov/cgi-bin/JK) and all cutoff values for separating the groups with high and low expression were determined using the online database algorithm (29). A good prognosis of neuroblastoma patients was considered to be associated with better survival.

### Soft agar clonogenic assay and sphere formation assay

Cells were mixed in 0.3% Noble agar (Sigma-Alrdich) in DMEM supplemented with 10% FBS and plated at 4,000 cells/well into 6-well plates, which contained a solidified bottom layer composed of 0.6% Noble agar in the same growth medium. At 14 days, colonies were stained with 5 mg/ml MTT and photographed (Olympus, IX71; Olympus). For sphere formation assays, cells were plated at 4,000 cells/well in serum-free DMEM, and supplemented with 20 ng/ml epidermal growth factor and basic fibroblast growth factor (Invitrogen Life Technologies) in Matrigel ultra-low attachment plates (Thermo Fisher Scientific, Pitsburgh, PA, USA). Spheres that arose within 1–2 weeks were counted.

### In vivo tumorigenic assay

For the tumorigenic assays, six female NOD/SCID mice (4 weeks old) were used and were maintained under SPF conditions. For the tumorigenic assays, the mice were randomly divided into two groups, control group and GATA3-overexpressing group. Mice were injected subcutaneously in both flanks with 1×10^7^ SK-N-SH cells or SK-N-SH-GATA3 cells in 200 μl DMEM. At one week following the injection of tumor cells, tumor growth was estimated using calipers, and tumor volume was calculated using the formula 4/3πr^3^, where r is the radius of the tumor. Tumors were removed and weighed following three weeks of tumor growth. The present study was approved by the Institutional Animal Care and Use Committee of Southwest University (Chonqing, China).

### Statistical analysis

Data are presented as the mean ± standard deviation. Two-tailed Student’s t-test was conducted for paired samples and was performed using GraphPad Prism version 6.0 software (GraphPad Software, Inc., La Jolla, CA, USA). P≤0.05 was considered to indicate a statistically significant difference.

## Results

### High GATA3 expression predicts poor survival in neuroblastoma patients

**Figure 1:**
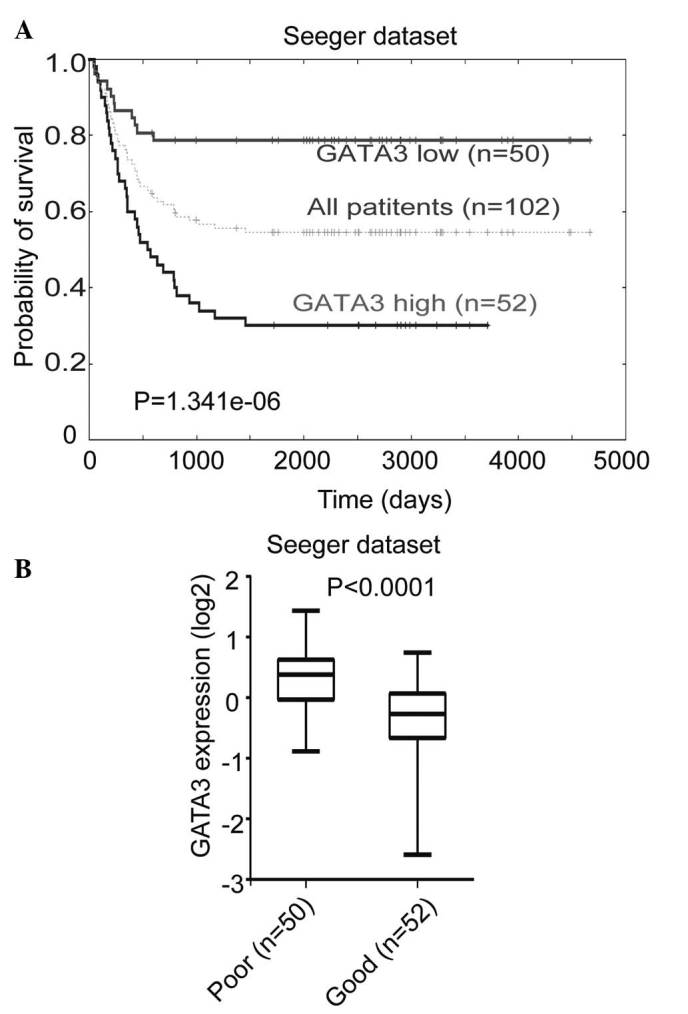
Association between GATA3 expression and survival in patients with neuroblastoma. (A) Kaplan-Meier analysis of progression-free survival for the Seeger database, with the log rank test P-value indicated. A cutoff value of 0.0365 was used to separate the patients into high and low GATA3 expression groups. (B) Box plot of GATA3 expression levels in tumors from groups of patients with good and poor prognoses.

The correlation between GATA3 expression levels and prognosis in primary neuroblastoma was investigated using the Seeger microarray dataset, which is available from the online Oncogenomics database. This dataset includes a cohort of 102 neuroblastoma patients with metastatic tumors lacking MYCN amplification (30). Kaplan-Meier analysis of progression-free survival for the Seeger dataset showed that low GATA3 expression was associated with a good prognosis, whereas high GATA3 expression was associated with a poor outcome (Fig. 1A). Furthermore, a box plot of GATA3 expression levels in tumors from patients with either a good or a poor prognosis demonstrated the same result (Fig. 1B). This analysis indicated that GATA3 is a prognostic marker in neuroblastoma, which is independent of the status of MYCN amplification.

### GATA3 is commonly expressed in neuroblastoma cells

**Figure 2:**
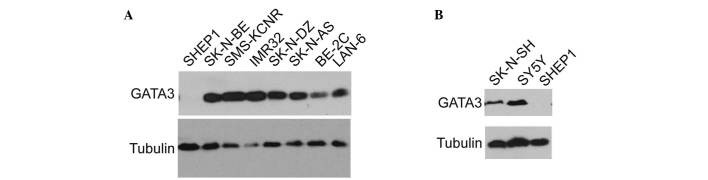
Expression of GATA3 in various neuroblastoma cell lines. (A) Western blot analysis of GATA3 expression in eight neuroblastoma cell lines. (B) Western blot analysis of GATA3 expression in SK-N-SH, SK-N-SY5Y and SHEP1 cell lines. α-tubulin levels were used as a loading control.

The expression of GATA3 in various neuroblastoma cell lines was examined. GATA3 was found to be widely expressed in the majority of neuroblastoma cell lines (Fig. 2A), including BE (2)-C, IMR32, SK-N-DZ, SK-N-AS, and SK-N-BE, which are malignant cell lines. The expression of GATA3 was also investigated in the SHEP1 cell line, which is a benign neuroblastoma cell line with a highly differentiated status (31,32). The results indicated that GATA3 may be associated with the degree of neuroblastoma differentiation and the consequent prognosis. The SK-N-SH cells were a group of mixed cells which were isolated into SY5Y and SHEP1 in different conditions (33), and SY5Y is a type of malignant cell comparing with SHEP1 (32). As hypothesized, GATA3 expression was relatively high in the SY5Y and SK-N-SH cell lines, but there was no detectable expression in the SHEP1 cell line (Fig. 2B). This suggests that GATA3 may be used as a prognostic marker in neuroblastoma.

### GATA3 is associated with neuronal differentiation in neuroblastoma cells

**Figure 3:**
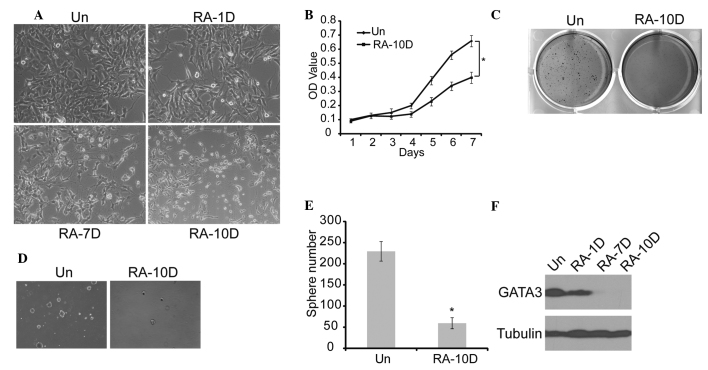
Association between GATA3 expression and neuronal differentiation in neuroblastoma cells. (A) Morphological examination of SK-N-SH cells treated with RA or DMSO (magnification, ×20). (B) SK-N-SH cells were treated with RA or DMSO, and cell proliferation was analyzed with an MTT assay. (C) SK-N-SH cells were plated at 4×10^3^ cells per well in six-well culture plates. At days 14 to 21 soft agar colonies grew from the cells treated with DMSO. Cells treated with RA were observed to give rise to small and scanty colonies in soft agar. (D) and (E) SK-N-SH cells were plated at 4×10^3^ cells per well in Matrigel ultra-low attachment plates. At days 14 to 21, spheres grew from cells treated with DMSO, and were recorded. (F) Western blot analysis of GATA3 expression in SK-N-SH cells treated with RA or DMSO (magnification, ×10). α-tubulin levels are shown as a loading control. Data in (B) and (E) are presented as the average obtained from three independent experiments. Error bars, represent standard deviation. ^*^P≤0.05. RA, retinoic acid; Un, untreated; DMSO, dimethyl sulfoxide; RA-(1D, 7D and 10D), one day, seven days and ten days following RA treatment, respectively; OD, optical density.

Since RA is commonly used to induce neuronal differentiation in neuroblastoma (34), SK-N-SH cells were treated with RA for 10 days. On examination the cells displayed morphological features of neuronal differentiation, such as small and rounded cell bodies, scant cytoplasm, and extensive neurite-like processes (Fig. 3A). Furthermore, MTT, sphere formation and soft agar analyses showed that RA treatment resulted in the suppression of cell proliferation, tumorigenicity and self-renewal of neuroblastoma cells (Fig. 3B–E). The GATA3 expression in this process was measured, and the results showed that RA induction led to marked downregulation of GATA3 expression with time (Fig. 3F). These findings suggest that RA-induced neuronal differentiation is accompanied by GATA3 downregulation, which leads to weakened self-renewal of neuroblastoma cells.

### GATA3 promotes proliferation and tumorigenicity of neuroblastoma cells

**Figure 4:**
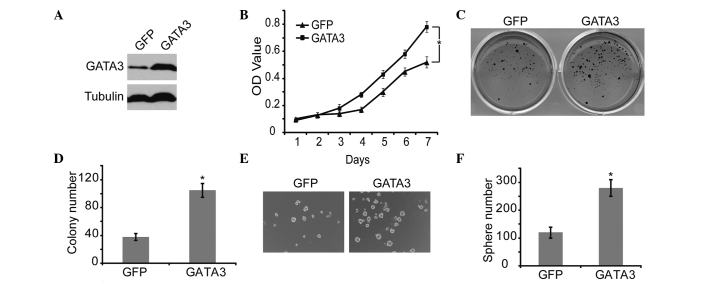
Effect of GATA3 overexpression on the proliferation and self-renewal of neuroblastoma cells. (A) Western blot analysis of GATA3 expression in SK-N-SH cells with GFP or GATA3 overexpression. α-Tubulin levels were used as a loading control. (B) SK-N-SH cells with GFP or GATA3 overexpression were analyzed for cell growth curve with an MTT assay. (C) SK-N-SH cells with GFP or GATA3 overexpression were plated at 4×10^3^ cells per well in six-well culture plates. At days 14 to 21, soft agar colonies grew from cells with GFP or GATA3 overexpression. Cells with GATA3 overexpression were observed to give rise to larger and more colonies in soft agar. (D) Colonies >0.5 mm or that contained >50 cells were recorded. (E) and (F) SK-N-SH cells with GFP or GATA3 overexpression were plated at 4×10^3^ cells per well in Matrigel ultra-low attachment plates. At days 14 to 21, spheres grew and were recorded (magnification, ×10). Data in (B), (D) and (F) are presented as the average obtained from three independent experiments. Error bars represent the standard deviation. ^*^P≤0.05. GFP, green fluorescent protein; OD, optical density.

To confirm the correlation between GATA3 and self-renewal of neuroblastoma cells, GATA3 was overexpressed in neuroblastoma cells, using GFP as a control (Fig. 4A). GATA3 significantly increased cell proliferation, which was verified by MTT analysis (Fig. 4B). In addition, GATA3 markedly upregulated the self-renewal ability, including the colony forming and sphere forming capability, of neuroblastoma cells (Fig. 4C and 4F). These results demonstrated that high expression of GATA3 is associated with increased self-renewal and cell proliferation in neuroblastoma cells.

**Figure 5:**
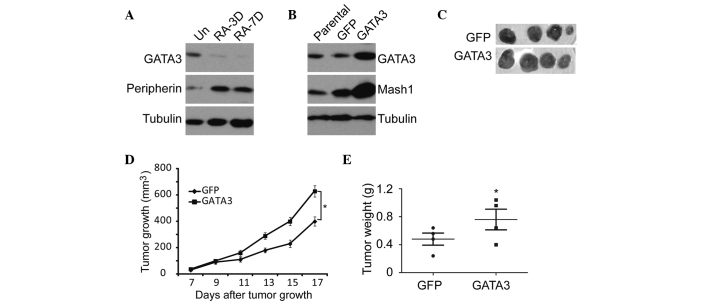
Effect of GATA3 overexpression on the tumorigenicity of neuroblastoma cells. (A) Western blot analysis of GATA3 and peripherin expression in SK-N-SH cells treated with RA or DMSO. (B) Western blot analysis of GATA3 and Mash1 expression in SK-N-SH cells with GFP or GATA3 overexpression. α-tubulin levels were used as a loading control. (C) Tumor growth in NOD/SCID mice injected with indicated SK-N-SH cells. (D) Scatter plot of xenograft tumor weight with horizontal lines indicating the mean per group. (E) Xenograft tumor volume was measured using calipers. ^*^P≤0.05. Scale bar=5 cm. GFP, green fluorescent protein; Un, untreated; RA-3D/RA-7D, three and seven days, respectively, following retinoic acid administration.

Furthermore, the RA-induced neuroblastoma differentiation was accompanied by GATA3 downregulation and the upregulation of peripherin, a neuronal differentiation marker (35) (Fig. 5A). GATA3 upregulation led to a significant increase in the expression of Mash1 (Fig. 5B), which is a potential stem cell or progenitor cell marker (36,37). Similar results were obtained from the *in vivo* tumorigenicity analysis using the SK-N-SH neuroblastoma cells. Overexpression of GATA3 in SK-N-SH cells significantly enhanced tumor growth and development in NOD/SCID mice (Fig. 5C–E). These data indicate that GATA3 is not only a prognostic marker, but also an important mediator of cell proliferation and differentiation.

## Discussion

The current study provided a number of lines of evidence to support the hypothesis that GATA3 acts as an important mediator of neuroblastoma differentiation. GATA3 was shown to be expressed at significantly lower levels following RA-induced differentiation. Overexpression of GATA3 expression significantly increased cell growth and self-renewal in neuroblastoma cells. Furthermore, RA-induced neuronal differentiation resulted in the upregulation of peripherin, a neuronal differentiation marker, and the downregulation of GATA3. In turn, GATA3 overexpression increased the expression of a marker of a self-renewal marker, Mash1. These results suggest a possible molecular mechanism linking neuronal differentiation and self-renewal. Using a gene expression dataset of 102 metastatic neuroblastoma tumors, it was shown that high GATA3 expression is a prognostic marker of poor outcome, which supported the results of the other experiments.

MYCN is an important oncogene in the pathogenesis of neuroblastoma (38), and is known to regulate various cellular processes, including cell growth, cell proliferation, cell differentiation and apoptosis (39,40). The oncogene MYCN was originally identified in neuroblastoma cells (41,42), and it has been reported as a prognostic marker in patients with neuroblastoma (43). Amplification of MYCN occurs in 22% of cases of neuroblastoma and is associated with advanced stages of this disease and a poor prognosis (7). N-myc is currently the only marker commonly used in the diagnosis of neuroblastoma. It is therefore necessary to identify further genetic markers for neuroblastoma. The current study showed that high GATA3 expression was correlated with poor survival in patients with neuroblastoma. Low expression of GATA3 was associated with a high degree of differentiation, indicating that GATA3 may be of use as a prognostic marker in neuroblastoma.

Neuroblastoma originates from precursor neuroblasts of the sympathetic nervous system, and is characterized by a unique capacity for complete spontaneous regression, at least partly through the process of neuronal differentiation (8). In clinical practice, patients with advanced neuroblastoma may be successfully treated by the administration of RA, which induces tumor cells to differentiate and leads to growth inhibition (9,11,44). In the current study, neuronal differentiation, induced by RA, was accompanied by GATA3 downregulation in neuroblastoma cells, whereas upregulation of GATA3 was associated with increased self-renewal and proliferation of neuroblastoma cells. In conclusion, the present results confirmed that GATA3 has an important function in neuroblastoma differentiation and proliferation. Therefore, GATA3 may be useful as a prognostic marker in patients with neuroblastoma, and may also serve as a potential therapeutic target for neuroblastoma.
